# Spontaneous expulsion of 10 years retained intrathoracic foreign body (bullet) from left main bronchus: A case report

**DOI:** 10.1016/j.amsu.2022.104640

**Published:** 2022-09-14

**Authors:** Abdullahi Abdi Ahmed, Ali Mohamed Warsame, Hassan Özdamir, Yigit Yilmaz, Najib Mohamed Salad, Yahye Garad Mohamed

**Affiliations:** a Thoracic Surgery Department, Mogadishu Somalia Turkey, Recep Tayyip Erdogan Training and Research Hospital, Mogadishu, Somalia; b General Surgery Department, Mogadishu Somalia Turkey, Recep Tayyip Erdogan Training and Research Hospital, Mogadishu, Somalia; c Radiology Department, Mogadishu Somalia Turkey, Recep Tayyip Erdogan Training and Research Hospital, Mogadishu, Somalia

**Keywords:** Intrathoracic foreign body, Spontaneous expulsion, Intrathoracic, A case report

## Abstract

A foreign body lodged in the tracheobronchial tree is a serious and common medical emergency that can have serious and perhaps lethal consequences.

A few cases have been reported in the literature for the last century with gunshot wounds to the chest that were handled non-operatively and finally expelled the bullet on their own.

We present a case of a hemodynamically stable 50-year-old male with a 10-year-old penetrating thoracic gunshot wound, with the bullet found in the left main bronchus on computed tomography (CT) scan upon admission. Further examination found no evident erosive injuries, such as hemoptysis, but he did have empyema and required a thoracotomy for decortication. Shortly after discharge, he coughed out a bullet into the floor, which is why our case is so intriguing.

This case demonstrated that a bronchial foreign body is seldom spontaneously expelled.

## Highlights

• The expulsion of a foreign body from the left major bronchus as in our case is also extremely rare.
• If a foreign body is found in the thoracic cavity, it can be removed using a flexible or rigid bronchoscope, video-assisted thoracoscopy (VATS), or open surgery.
• Spontaneous expulsion of the intrabronchial foreign body prevents the patient from surgical removal of a foreign body, but it also poses a significant risk of intratracheal foreign body lodgement and thoracic empyema, as in our case.

## 1 Introduction

A foreign body stuck in the tracheobronchial tree is a severe and common medical emergency that can have significant and perhaps fatal effects [1]. Because spontaneous ejection of intrapulmonary foreign materials occurs so seldom, Jackson suggested in 1921 that removal should be conducted early to minimise further difficulties [2,3], hence spontaneous expulsion of foreign body bronchus is incomprehensible and extremely unusual. Intrathoracic foreign bodies are classified into intrapulmonary and extrapulmonary [4]. Extrapulmonary foreign bodies are most commonly caused by penetrating traumas like bullets or shrapnel. Because they are enclosed by fibrous tissue and hence have no tendency to harm adjacent tissues, they are frequently asymptomatic.

## 2 Case report

**Fig. 1:**
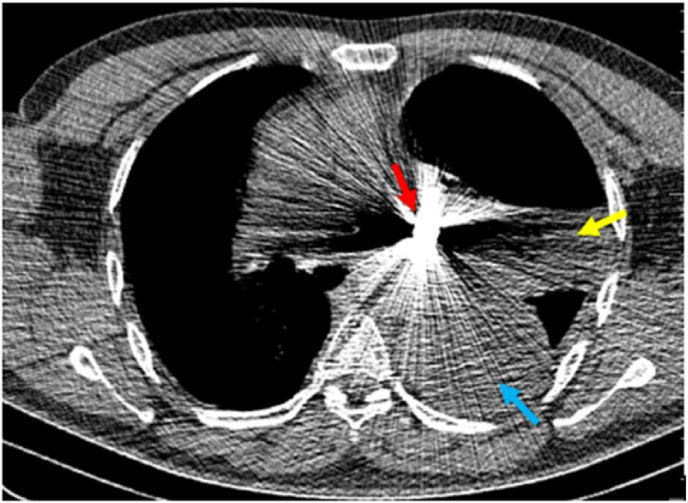
Non-contrast CT chest demonstrating impacted foreign body (Bullet) (red arrow) in left hemithorax especially left main bronchus with resultant left lung atelectasis (yellow arrow) and thoracic empyema (blue arrow). (For interpretation of the references to colour in this figure legend, the reader is referred to the Web version of this article.)

**Fig. 2:**
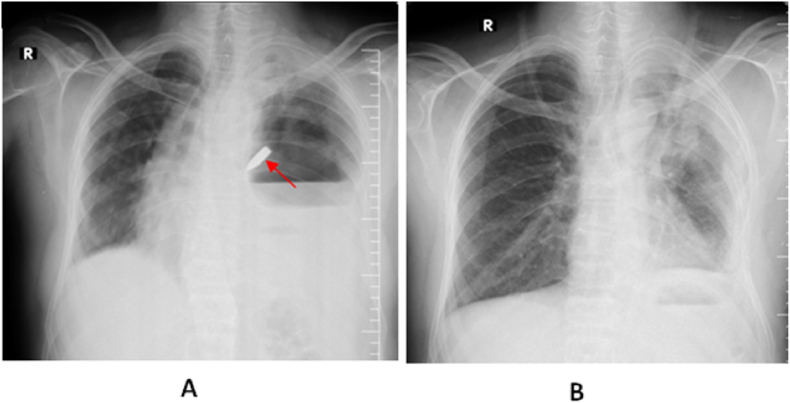
**A**- 3-mm radiopaque foreign body (red arrow) is seen in the left main bronchus on a chest radiograph. The left hemithorax also has an air-fluid level comparable to empyema.**B**- x-ray after spontaneous bullet expectoration. (For interpretation of the references to colour in this figure legend, the reader is referred to the Web version of this article.)

**Fig. 3:**
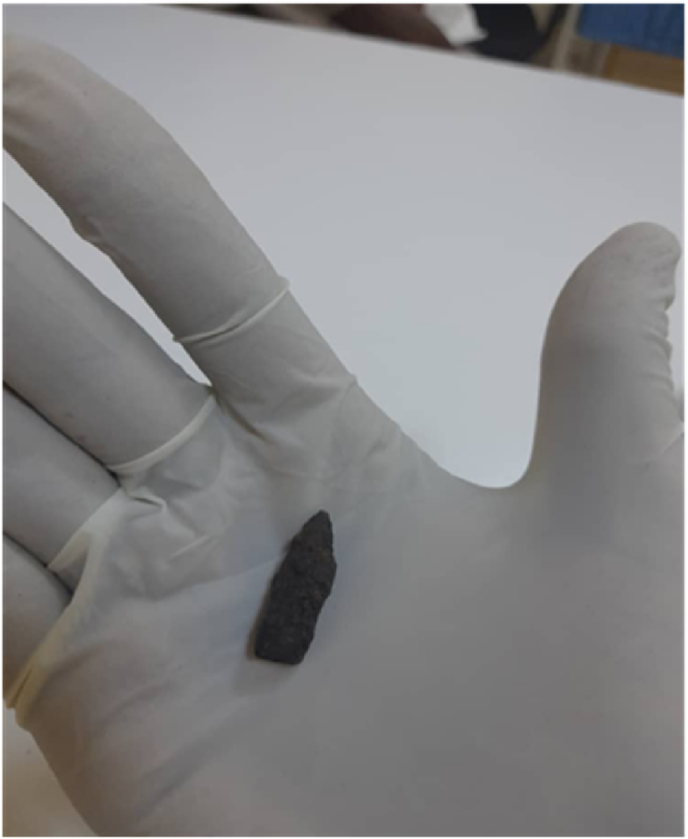
The intact bullet that was spontaneously expectorated.

We present a case of a 50-year-old male who has been asymptomatic for nearly ten years after a penetrating chest bullet lodged in his left main bronchus. When he arrived at the emergency hospital, his major concerns were chest discomfort and breathing difficulty. He seemed alert, attentive, and anxious at the emergency department. The patient experienced tachycardia and tachypnea throughout the physical examination. After a chest CT revealed a thoracic empyema and a foreign body in the left main bronchus, a new chest tube was placed for drainage, A culture and sensitivity were also done, and the patient was given the proper antibiotic. Within two weeks, a thoracotomy was performed for decortication. The patient was discharged from the hospital after 15 days. But after three months he coughed up a bullet on his own (see Fig. 1, Fig. 2, Fig. 3).

## 3 Discussion

After the early post-injury phase, retained intrathoracic foreign bodies caused by penetrating chest damage seldom elicit symptoms. Foreign bodies often remain in a fixed place throughout the patient's life after scarring and healing. They only occasionally erode onto nearby structures [5]. Recurrent pneumonia, empyema, hemoptysis, and endovascular embolization of the bullet are all possible complications. If a bullet is found in the thoracic cavity, it can be removed using a flexible or rigid bronchoscope, video-assisted thoracoscopy (VATS), or open surgery [6]. But our case was asymptomatic for about ten years. The bullet remained intrathoracic and did not show signs and symptoms of erosion such as hemoptysis. He underwent a thoracotomy due to empyema, and the bullet was not found because it was in the left main bronchus. Another interesting characteristic is that, for obvious anatomical reasons, a foreign body should be simpler to lodge into and remove from the right major bronchus. But in case, a heavy foreign body was expelled out from the left main bronchus, and that too by force of cough from the lung whose capacity was already compromised due to collapse and consolidation, which is again an unusual and unlikely occurrence [7]. The expulsion of a foreign body from the left major bronchus as in our case is also extremely rare according to our experience in the thoracic surgery field. Up to date, only four cases have been published of patients sustaining gunshot wounds to the chest, managed nonoperatively, who eventually expectorated the bullet. In each case, the patient presented with stable vital signs, one bullet wound, and a visible bullet in the chest on a radiograph [8] was the first described patient in 1917.

The spontaneous expulsion of foreign body bronchus is an uncommon occurrence, with just a few cases documented in the literature. Gupta and Sood reported two cases: one involving a 2-year-old male kid who spontaneously ejected a cycle tube metallic valve from the right bronchus four days after inhalation, and the other involving a 20-year-old guy who suddenly expelled a four-anna copper coin from the trachea [9].

This work has been reported in line with the SCARE 2020 criteria [10].

## 4 Conclusion

This case highlighted the rarity of spontaneous expulsion of a sharp metallic foreign material from the bronchus. On the one hand, it prevents the patient from surgical removal of a foreign body, but it also poses a significant risk of intratracheal foreign body lodgement and thoracic empyema, as in our case.

## Ethical approval

Ethical approval.

Ethical approval was waived by the ethical committee of Mogadishu Somali Turkey, Recep Tayyip Erdogan Training and Research Hospital.

## Please state any sources of funding for your research

Funding was received.

## Author contributions

Author contribution/CRediT author statement.

o AAA- Conceived the idea, Corresponding author, write the manuscript, AMW- Literature Review, and Editing, HÖ- Supervision, and Data Validation, YY- Supervision, and Data Validation, NMS- Collected the data, and Editing, YGM-reviewed and revised the manuscript for intellectual content critically. All authors approved the final version of the manuscript.

## Registration of research studies

1. Name of the registry: N/A.

2. Unique identifying number or registration ID: N/A.

3. Hyperlink to your specific registration (must be publicly accessible and will be checked): N/A.

## Guarantor

Abdullahi Abdi Ahmed.

Phone. +252618009917.

Email. drkacaaye@gmail.com.

## Consent

Written informed consent was obtained from the patient for publication of this case report and accompanying images. A copy of the written consent is available for review by the Editor-in-Chief of this journal on request.

## Availability of data and materials

The data that support the findings of this study are available in Mogadishu Somali Turkey, Recep Tayyip Erdogan Training and Research Hospital information system. Data is however allowed to the authors upon reasonable request and with permission of the education and research committee.

## Provenance and peer review

Not commissioned, externally peer-reviewed.

## Abbreviations

N/A.

## Please state any conflicts of interest

This manuscript has not been submitted to, nor is it under review at, another journal or other publishing venue.

The authors have no affiliation with any organization with a direct or indirect financial interest in the subject matter discussed in the manuscript.
